# Tumor-associated macrophages predict prognosis in diffuse large B-cell lymphoma and correlation with peripheral absolute monocyte count

**DOI:** 10.1186/s12885-019-6208-x

**Published:** 2019-11-06

**Authors:** Yan-Li Li, Zhi-Hu Shi, Xian Wang, Kang-Sheng Gu, Zhi-Min Zhai

**Affiliations:** 1 Department of Pathology, Anhui Medical University, Hefei, Anhui 230032 People’s Republic of China; 2 Department of Pathology, The First Affiliated Hospital of Anhui Medical University, Hefei, Anhui 230022 People’s Republic of China; 3 Department of Pathology, Anhui Ji Min Cancer Hospital, Hefei, Anhui 230012 People’s Republic of China; 4 Department of Pathology, The Second Affiliated Hospital of Anhui Medical University, Hefei, Anhui 230601 People’s Republic of China; 5 Department of Oncology, The First Affiliated Hospital of Anhui Medical University, Hefei, Anhui 230022 People’s Republic of China; 6 Department of Hematology, The Second Affiliated Hospital of Anhui Medical University, Hefei, Anhui 230601 People’s Republic of China

**Keywords:** Diffuse large B-cell lymphoma, Tumor-associated macrophages, Absolute monocyte count, Prognosis

## Abstract

**Background:**

Diffuse large B-cell lymphoma (DLBCL) is characterized by its clinical and biological heterogeneity. The clinical prognostic implications of tumor-associated macrophages (TAMs) in DLBCL remain controversial and the correlation between TAMs and peripheral absolute monocyte count (AMC) has not yet been elucidated.

**Methods:**

In 221 untreated, newly diagnosed patients with DLBCL, we evaluated the prognostic value of TAMs using immunohistochemical analysis, as well as the association of TAMs and AMC.

**Results:**

We found that high CD68 or high CD163 expression was correlated with clinicopathological characteristics, high CD163 expression was an adverse predictor for both overall survival (OS) [hazard ratio (HR) = 2.265, *P* = 0.005] and progression- free survival (PFS) (HR = 1.925, *P* = 0.017) in patients with DLBCL. Patients with high CD68 or high CD163 expression had significantly poorer OS and PFS than those with low CD68 or low CD163 expression, respectively (CD68: OS: *P*<0.001, PFS: *P*<0.001; CD163: OS: *P*<0.001, PFS: *P*<0.001), even in the rituximab era. Moreover, high-risk patients could be further identified by the expression of CD68 or CD163, especially in those classified as low/intermediate risk by International Prognostic Index (IPI). Furthermore, the significant positive correlation was also detected between CD68 expression or CD163 expression and AMC (*r* = 0.256, *P*<0.001; *r* = 0.303, *P*<0.001).

**Conclusions:**

Patients with high expression of TAMs tend to have poorer OS and PFS, even in the rituximab era, and have positive correlation with AMC. Therefore, the peripheral AMC is a useful prognostic marker reflecting the status of the tumor microenvironment (TME) in DLBCL.

## Background

Diffuse large B-cell lymphoma (DLBCL) is the most common adult non-Hodgkin lymphoma (NHL), accounting for 30–40%. Although aggressive, it can be cured in 60–70% of cases after first-line immunochemotherapy. Nevertheless, 30–40% of cases will experience recurrence or refractory disease after initial response, which will dramatically reduce their survival time. These patients remain a challenging therapeutic problem, and more targeted and personalized approach is an important goal for them, which is still being explored [1].

In the past decades, the view that tumors mainly consist of tumor cells has changed, and tumors are now thought to have organoid structures. In addition to tumor cells, there are many other tumor-infiltrating stromal cells, such as fibroblasts, immune cells, vascular endothelial cells and mesenchymal stem cells, which interact closely with tumor cells, constituting the tumor microenvironment (TME) [2]. TME influences initiation, growth and metastasis of tumors, including DLBCL.

Tumor-associated macrophages (TAMs), as the intrinsic cellular components of TME, involve in tumor proliferation, invasion, angiogenesis, metastasis and suppression of anti-tumor immunity in many tumors [3]. In general, macrophages (MPs) often have two kinds of phenotypes because of different activation states. One is “classically” activated M1 phenotype with antitumor activity, the other is “alternatively” activated M2 phenotype with tumor-promoting activity [4]. So far, the clinical prognostic significance of TAMs in DLBCL remains controversial mainly due to difference in the method used to evaluate MPs as well as types of treatment given [5–13].

Another known prognostic marker in DLBCL is the peripheral absolute monocyte count (AMC), and elevated AMC levels have been reported to correlate with poor prognosis in DLBCL patients [14–20]. It is widely known that the majority of TAMs are derived from peripheral monocytes, however, the correlation between TAMs and AMC has not yet been elucidated in DLBCL. Therefore, in this study, we evaluated the predictive value of TAMs and clarified the correlation between TAMs and AMC in DLBCL patients.

## Methods

### Ethics statement

This study was carried out in accordance with the principles of the Helsinki Declaration and approved by the ethics committee of the first affiliated and the second affiliated hospitals of Anhui Medical University. Each patient had signed informed consent.

### Patients

two hundred twenty-one untreated, newly diagnosed patients with DLBCL were recruited between 2004 and 2015 from the First Affiliated Hospital and the Second Affiliated Hospital of Anhui Medical University, excluding immunodeficiency, transformed DLBCL, primary central nervous system DLBCL, EBV+ DLBCL, HHV8+ DLBCL and primary cutaneous DLBCL, leg type. For diagnosis and prognostic purposes, the current World Health Organization classification [21] and International Prognostic Index (IPI) [22] were used. All patients were treated with CHOP (cyclophosphamide, hydroxydaunorubicin, vincristine, prednisone) or R-CHOP (rituximab- cyclophosphamIde, hydroxydaunorubicin, vincristine, prednisone) every 3 weeks for 3 to 8 cycles as first-line therapy, and follow-up in the hospitals.

### Blood sample analysis

The AMC at diagnosis was obtained from routine automated complete blood count (CBC), and the cut-point for the AMC at diagnosis was 460/μl based on our previous data [14].

### Immunohistochemistry

two hundred twenty-one specimens for immunohistochemical analysis were formalin-fixed and paraffin- embedded. 4-um-thick paraffin sections and monoclonal antibodies against human CD68 (1:400 dilution, KP1, OriGene, USA) and CD163 (1300 dilution, 10D6, OriGene, USA) were used in the study. The specimen sections were deparaffinized in xylene and rehydrated in a series of grade alcohols. They were then protreated in citric acid antigen retrieval solution (pH 6.0) using heat-induced epitope retrieval technique. After inhibiting internal peroxidase activity with 3% hydrogen peroxide, the sections were incubated with anti-CD68 antibody and anti-CD163 antibody overnight at 4 °C. The slides were then incubated with HRP-conjugated goat anti- mouse IgG secondary antibody for 10 min at 37 °C. Finally, the sections were visualised by DAB solution (DAKO, Carpinteria, CA, USA) and counterstained with haematoxylin (DAKO). CD68 positive staining was observed in cytoplasm of MPs, and CD163 positive staining was observed in membrane of MPs. Two professional pathologists who did not know the clinical data analysed all stained sections by quantitative method. The average count of five high-power fields (HPF) (× 400, an area of view 0.24mm^2^) for each slice was obtained by an Olympus BX51TF microscope (Olympus, Japan).

### Statistical analysis

Receiver operating characteristics (ROC) analysis and area under the curve (AUC) were used to determine the optimal cut-point for the TAMs [9, 12]. The correlation between TAMs and clinical characteristics or AMC used the chi-square test or Fisher’s exact test. Univariate and multivariate analyses using Cox proportional hazards model were performed to explore whether TAMs was prognostic factors for survival when in combination with other clinicopathological factors. Overall survival (OS) was defined as the time from diagnosis until death or the last follow-up.Progression-free survival (PFS) was defined as the time from diagnosis until disease progression, relapse, death of any cause or the last follow-up. OS and PFS were estimated using the Kaplan-Meier method and compared using the log-rank test. *P* values<0.05 were determined to be statistically significant. Statistical analysis was performed using SPSS 17.0 software (SPSS Inc., Chicago, IL, USA).

## Results

### Immunohistochemical CD68 and CD163 intensity and cut-points for CD68 + cells and CD163 + cells

**Fig. 1:**
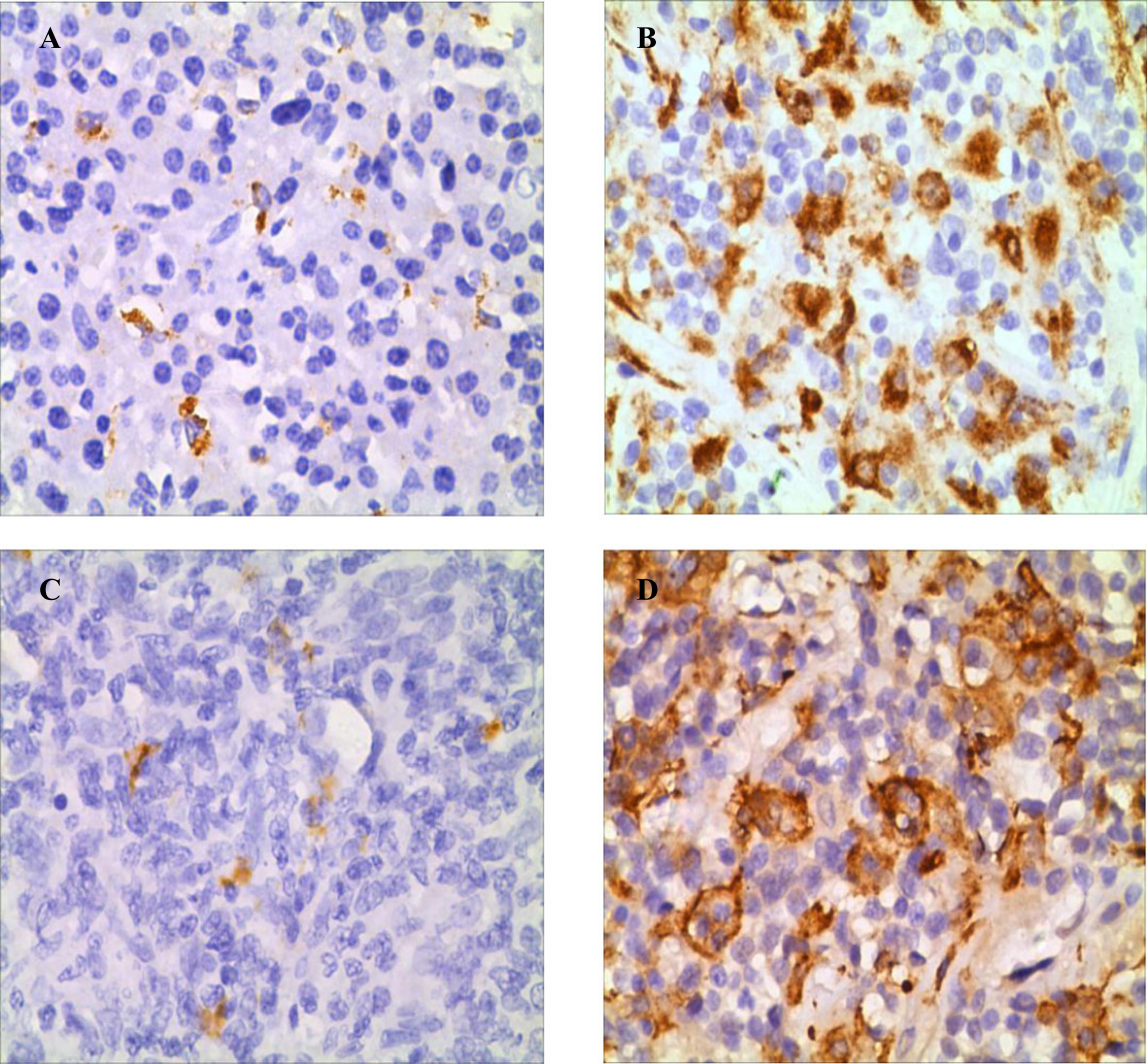
CD68 and CD163 expression in DLBCL tissues (×400). **a** low CD68 expression, **b** high CD68 expression, **c** low CD163 expression, **d** high CD163 expression

By immunohistochemical analysis, in tumor tissue, the median level of CD68 + cells/ HPF was 27 (range, 7–83), and the median level of CD163 + cells/HPF was 17 (range, 2–78). Based on survival information (death/survival at 5 years after diagnosis), the ROC curves and AUC were used to determine their cut-points. The most optimal cut-point of CD68 + cells was 33/HPF, with an AUC value of 0.706 (95%CI, 0.638–0.774, *P*<0.001) (Additional file 1: Figure S1A); the most optimal cut-point of CD163+ cells was 19/HPF, with an AUC value of 0.729 (95%CI, 0.663–0.795, *P*<0.001) (Additional file 1: Figure S1B). Of 221 DLBCL patients, 138 (62.4%) were categorized as low CD68 expression (CD68 + cells<33/HPF) and 83 (37.6%) as high CD68 expression (CD68 + cells≥33/HPF) (Fig. 1a, b), 121 (54.8%) were categorized as low CD163 expression (CD163 + cells<19/HPF) and 100 (45.2%) as high CD163 expression (CD163 + cells≥19/HPF) (Fig. 1c, d).

### The clinicopathological characteristics according to CD68 or CD163 expression and correlation of CD68 or CD163 expression with AMC

The median age of 221 DLBCL patients was 56 years (range, 13–84 years). The median follow-up time after diagnosis was 42 months (range, 3–118 months). The clinico- pathological characteristics are showed in Table 1. High CD68 expression was significantly correlated with poor ECOG-PS (*P*<0.001), more extranodal sites of disease (*P* = 0.014), III/IV Ann Arbor stage (*P*<0.001), elevated LDH (*P*<0.001), 3–5 IPI score (*P*<0.001) and high AMC (*P*<0.001), and high CD163 expression was significantly correlated with poor ECOG-PS (*P* = 0.018), more extranodal sites of disease (*P* = 0.026), III/IV Ann Arbor stage (*P* = 0.004), elevated LDH (*P*<0.001), 3–5 IPI score (*P*<0.001) and high AMC (*P*<0.001) (Table 1). The frequencies of CD68 and CD163 expression level significantly increased gradually accompanied with IPI score (CD68: *P*<0.001, Additional file 1: Figure S2A; CD163: *P*<0.001, Additional file 1: Figure S2B).

**Table 1:** Patient’s demographics according to CD68 and CD163 expression

Characteristics	Patients		CD68 expression			CD163 expression		
	NO.	%	Low	High	*P*	Low	High	*P*
Gender								
Female	105	47.5	70	35	0.217	62	43	0.222
Male	116	52.5	68	48		59	57	
Age (years)								
≤ 60	130	58.8	87	43	0.100	73	57	0.617
>60	91	41.2	51	40		48	43	
ECOG-PS								
≤ 1	136	61.5	98	38	<0.001	83	53	0.018
>1	85	38.5	40	45		38	47	
Extranodal sites of disease								
≤ 1	176	79.6	117	59	0.014	103	73	0.026
>1	45	20.4	21	24		18	27	
Ann Arbor stage								
I/II	127	57.5	93	34	<0.001	80	47	0.004
III/IV	94	42.5	45	49		41	53	
LDH								
≤ normal	159	71.9	114	45	<0.001	103	56	<0.001
>normal	62	28.1	24	38		18	44	
IPI score								
0–2	154	69.7	114	40	<0.001	98	56	<0.001
3–5	67	30.3	24	43		23	44	
AMC								
<460/ul	118	53.4	88	30	<0.001	82	36	<0.001
≥ 460/ul	103	46.6	50	53		39	64	

*Abbreviations*: *ECOG-PS* Eastern Cooperative Oncology Group performance status, *LDH* lactate dehydrogenase, *IPI* International Prognostic Index, *AMC* absolute monocyte count

**Fig. 2:**
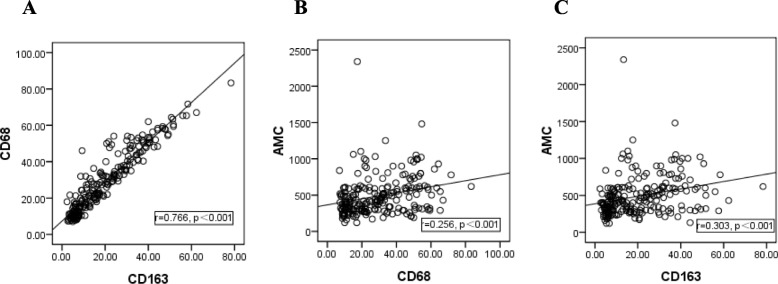
Spearman correlation analysis among CD68 expression, CD163 expression and AMC. **a** A positive correlation was found between CD68 expression and CD163 expression. A positive correlation was found between (**b**) CD68 expression or (**c**) CD163 expression and AMC

Significant positive associations were found between CD68 expression and CD163 expression (*r* = 0.766, *P*<0.001, Fig. 2a), between CD68 expression and AMC (*r* = 0.256, *P*<0.001, Fig. 2b), CD163 expression and AMC (*r* = 0.303, *P*<0.001, Fig. 2c) by spearman correlation analysis.

### High CD163 expression is an independent adverse predictor for survival

Univariate and multivariate analyses were performed to explore whether CD68 or CD163 expression was an independent prognostic factor for survival when in combination with other clinicopathological factors. Old age, poor ECOG-PS, more extranodal sites of disease, III/IV Ann Arbor stage, elevated LDH, 3–5 IPI score, high AMC, without rituximab therapy, high CD68 expression and high CD163 expression were all significant prognostic factors for poor survival by univariate analyses. The above-mentioned significant clinicopathological factors were entered into multivariate analysis (Table 2). As with III/IV Ann Arbor stage (OS: *P* = 0.047, PFS: *P* = 0.010), elevated LDH (OS: *P* = 0.005, PFS: *P* = 0.007), high AMC (OS: *P* = 0.011, PFS: *P* = 0.008), and without rituximab therapy (OS: *P* = 0.003, PFS: *P* = 0.010), we found that high CD163 expression was an independent adverse predictor for both OS and PFS (OS: *P* = 0.005, PFS: *P* = 0.017).

**Table 2:** Multivariate Cox regression analyses of potential prognostic factors for OS and PFS

Variables	OS		PFS	
	HR(95%CI)	*P*	HR(95%CI)	*P*
Age				
≤ 60 years/>60 years	0.987 (0.674–1.444)	0.946	0.835 (0.562–1.241)	0.373
ECOG-PS				
1/>1	1.126 (0.725–1.749)	0.596	1.037 (0.668–1.610)	0.872
Extranodal sites				
≤ 1/>1	1.019 (0.610–1.705)	0.941	1.038 (0.621–1.733)	0.888
Ann Arbor stage				
IorII/IIIorIV	1.620 (1.006–2.606)	0.047	1.862 (1.157–2.995)	0.010
LDH				
≤ normal/> normal	1.881 (1.214–2.916)	0.005	1.815 (1.173–2.810)	0.007
IPI score				
0–2/3–5	1.167 (0.562–2.424)	0.679	1.320 (0.631–2.760)	0.461
AMC				
<460/ul/≥460/ul	1.658 (1.124–2.446)	0.011	1.675 (1.142–2.457)	0.008
Treatment				
R-CHOP/CHOP	1.978 (1.269–3.082)	0.003	1.780 (1.149–2.756)	0.010
CD68				
low/high	1.237 (0.710–2.156)	0.453	1.369 (0.789–2.376)	0.264
CD163				
low/high	2.265 (1.280–4.008)	0.005	1.925 (1.125–3.291)	0.017

*Abbreviations*: *OS* overall survival, *PFS* progression-free survival, *HR* hazard ratio, *CI* confidence interval, *ECOG-PS* Eastern Cooperative Oncology Group performance status, *LDH* lactate dehydrogenase, *IPI* International Prognostic Index, *AMC* absolute monocyte count, *CHOP* cyclophosphamide hydroxydaunorubicin vincristine prednisone, *R-CHOP* rituximab-cyclophosphamidehydroxydaunorubicin vincristine prednisone

### Prognostic value of CD68 expression and CD163 expression in DLBCL patients

**Fig. 3:**
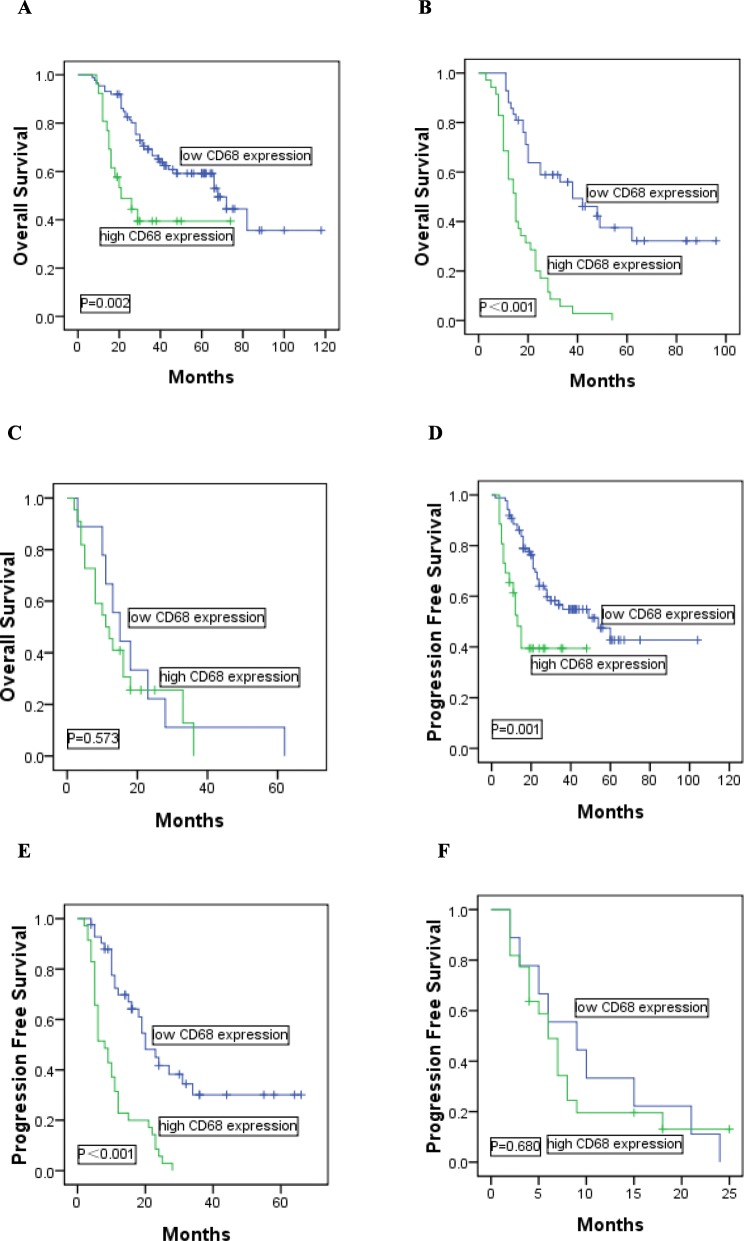
Kaplan-Meier analysis of OS and PFS according to the expression of CD68 in patients with DLBCL. Patients identified by the IPI score as (**a,d**) IPI 0-1, (**b,e**) IPI 2-3, (**c,f**) IPI 4-5 were further stratified into low CD68 expression or high CD68 expression groups, respectively

**Fig. 4:**
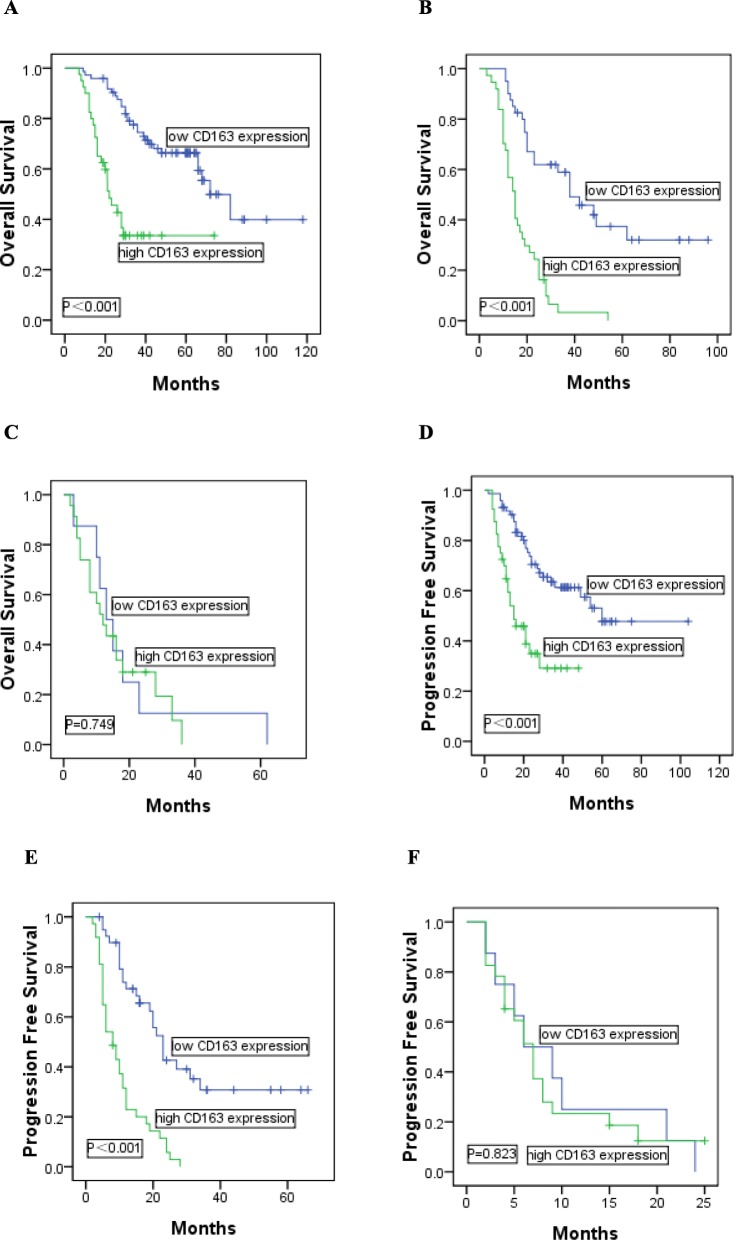
Kaplan-Meier analysis of OS and PFS according to the expression of CD163 in patients with DLBCL. Patients identified by the IPI score as (**a,d**) IPI 0-1, (**b,e**) IPI 2-3, (**c,f**) IPI 4-5 were further stratified into low CD163 expression or high CD163 expression groups, respectively

In comparison with low expression of CD68, shorter OS and PFS could be found in DLBCL patients with high expression of CD68 (median OS: 19 vs 41 months, *P*<0.001; median PFS: 11 vs 27 months, *P*<0.001). Meanwhile, DLBCL patients with high expression of CD163 had significantly poorer OS and PFS than those with low expression of CD163 (median OS: 19 vs 44 months, *P*<0.001; median PFS: 13 vs 28 months, *P*<0.001). We further examined whether CD68 or CD163 expression could identify high-risk patients in different IPI score subgroups including low risk (score = 0–1), intermediate risk (score = 2–3) and high risk (score = 4–5). In low risk group (*n* = 113), high-risk patients could be significantly identified by CD68 expression (median OS: 25 vs 46 months, *P* = 0.002, Fig. 3a; median PFS: 16 vs 32 months, *P* = 0.001, Fig. 3 d) or CD163 expression (median OS: 24 vs 50 months, *P*<0.001, Fig. 4a; median PFS: 17 vs 34 months, *P*<0.001, Fig. 4d). The intermediate risk (*n* = 77) patients were equally identified using CD68 expression (median OS: 17 vs 36 months, *P*<0.001, Fig. 3b; median PFS: 10 vs 22 months, *P*<0.001, Fig. 3e) or CD163 expression (median OS: 17 vs 37 months, *P*<0.001, Fig. 4b; median PFS: 10 vs 23 months, *P*<0.001, Fig. 4e). However, in high risk group (*n* = 31), CD68 or CD163 expression was not significantly predictive in the study (CD68: median OS: 13 vs 20 months, *P* = 0.573, Fig. 3c; median PFS: 8 vs 11 months, *P* = 0.680, Fig. 3f; CD163: median OS: 14 vs 19 months, *P* = 0.749, Fig. 4c; median PFS: 8 vs 10 months, *P* = 0.823, Fig. 4f).

**Fig. 5:**
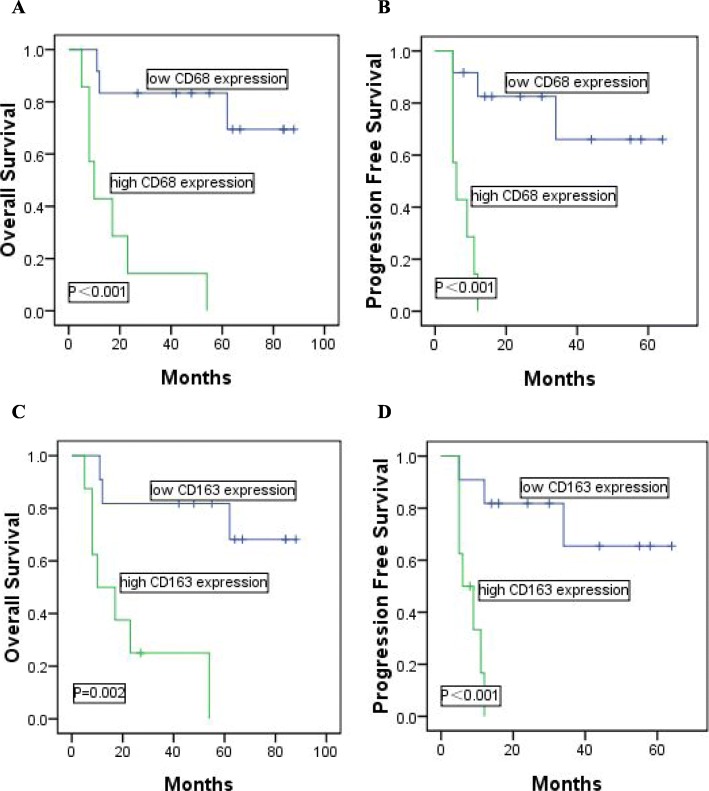
Patients treated with R-CHOP identified by IPI score as IPI 2-3 were further stratified based on (**a,b**) CD68 expression and (**c,d**) CD163 expression, respectively

In addition, in DLBCL patients who received R-CHOP (*n* = 59), high expression of CD68 or CD163 had significantly poorer OS and PFS than those with low expression of CD68 or CD163 (CD68: median OS: 23 vs 50 months, *P*<0.001; median PFS: 12 vs 33 months, *P*<0.001; CD163: median OS: 23 vs 54 months, *P*<0.001; median PFS: 14 vs 36 months, *P*<0.001). In intermediate risk group (*n* = 19), high-risk patients could be identified by CD68 or CD163 expression (CD68: median OS: 18 vs 54 months, *P*<0.001, Fig. 5a; median PFS: 8 vs 30 months, *P*<0.001, Fig. 5b; CD163: median OS: 19 vs 56 months, *P* = 0.002, Fig. 5c; median PFS: 8 vs 32 months, *P*<0.001, Fig. 5d).

## Discussion

The present study evaluated the clinical prognostic implications of TAMs in DLBCL, as well as the association with AMC. We used 2 markers to identify TAMs, CD68 and CD163, and found that high CD68 or CD163 expression was correlated with clinico- pathological characteristics, high CD163 expression was an adverse predictor for both OS and PFS. Patients with high CD68 or high CD163 expression had significantly poorer OS and PFS than those with low CD68 or low CD163 expression, respectively, even in the rituximab era. Moreover, high-risk patients could be further identified by the expression of CD68 or CD163, especially in those classified as low/intermediate risk by IPI. Furthermore, the significant positive correlation was also detected between TAMs and AMC.

A recognized hallmark of cancer is the non-resolving inflammation in TME. In addition to tumor cells, there are different stromal cells influencing the tumorigenesis, growth, metastasis and angiogenesis of tumors in TME, such as fibroblasts, leukocytes (including myeloid lineage cells and lymphoid lineage cells), vascular endothelial cells and mesenchymal stem cells. TAMs, generally thought to be more similar to M2- polarized MPs, are often prominent stromal cells that coordinate various factors in TME, and are also known as alternatively activated MPs, which are activated by helper T cell 2 (Th2) cytokines, such as interleukin (IL)-4, IL-10 and IL-13 [23]. In recent studies, TAMs in TME were found to correlate with clinical prognosis in hematologic neoplasms, particularly in Hodgkin’s lymphoma (HL) [24]. Nevertheless, its significance in DLBCL has thus far been controversial. The number of CD68 + cells did not exhibit significant prognostic value [13], and was found to have no significant relation with OS or PFS [5], whereas other researches showed that higher CD68 + cells tended to predict poorer prognosis [6, 8, 9] and worse treatment response [6, 11]. But recently, Nam et al. [8] and Riihijärvi S et al. [9] suggested that higher CD68 expression was correlated with better prognosis in DLBCL received rituximab in addition to other chemotherapy such as CHOP, and with poorer outcome when rituximab was not given. Our study results are in line with these findings [7, 8], suggesting that high CD68 expression was correlated with clinicopathological characteristics and had significantly poor OS and PFS, even in the rituximab era. In addition, it is generally believed that high CD163 expression predicts poor prognosis in DLBCL patients [7, 8, 10, 12, 13], and our results are consistent with those previous studies. The clinical prognostic significance of TAMs in DLBCL remains controversial possibly due to difference in clinical case-study method, patient populations, instrument (such as field of view) and method used to evaluate MPs as well as types of treatment given. In summary, TAMs may play an important role in promoting tumor growth, invasion and metastasis in TME of DLBCL, and CD163, as considered to be a marker relatively specific to M2 TAMs may serve as a better predictor than the pan-macrophage marker, CD68. There have been several studies on the role of TAMs in DLBCL. TAMs remodel extracellular matrix (ECM) remodeling and promote angiogenesis through producing matrix metalloprotein-9 (MMP-9), legumain, and vascular endothelial growth factor (VEGF) [25, 26]. Reactive oxygen species (ROS) from TAMs contributes to DLBCL progression and drug resistance by increasing the expression of CD44 [27]. TAMs express programmed death-1 ligand (PD-L1), which induces the apoptosis of T cells, involving immunosuppression and immune evasion [28, 29]. DLBCL cells can avoid phagocytosis through CD47/signal regulatory protein α (SIRPα) on TAMs pathway [30].

Monocytes and their progeny, promote tumor growth and angiogenesis, and involve in the suppression of host anti-tumor immunity. So more and more studies have found that elevation of peripheral monocytes is an adverse prognostic factor in many solid tumors [31–34], including DLBCL [14–20]. We have previously reported that peripheral AMC at diagnosis predicted outcome for DLBCL patients who received standard first-line regimens [14], elevation of AMC at the first relapse was an adverse prognostic factor for survive in relapsed/refractory DLBCL [15], and increased AMC tested during follow-up after standard first-line regimens was a risk factor for predicting recurrence of DLBCL [16]. Until now, few studies [34–37] have demonstrated that peripheral AMC significantly correlated with TAMs. However, to our knowledge, in DLBCL the correlation between AMC and TAMs has not been clarified. In our study, AMC was significantly positively correlated with TAMs in DLBCL patients. So, why peripheral blood monocytosis indicates poor prognosis in DLBCL, we deduce that monocytes differentiated into MPs after recruitment from peripheral blood to TME, then polarized into M2 phenotype thereby causing the progression of disease.

There are some limitations in the study. Firstly, as a retrospective study, patient selection might have been biased, and other unrecognized bias might have influenced the results. Secondly, the amount of cases is relatively small to prove conclusions. Thirdly, we used ROC curves and AUC to determine the best TAMs (CD68 + cells and CD163 + cells) cutoff value in our study, however, the cut-point for TAMs need investigation in other independent cohort.

## Conclusions

In conclusion, we confirm the prognostic implications of TAMs in DLBCL in a retrospective study. Our results show that higher levels of TAMs in TME tend to have poorer outcomes, even in the rituximab era. Our findings, in conjunction with other previous studies, establish TAMs to be important in the DLBCL TME. As far as we know, this study is the first to identify the correlation between TAMs and AMC in DLBCL. Therapeutic strategies to inhibit M2 polarization may, therefore, be a potential target for anticancer therapy in DLBCL.

## Supplementary information

**Additional file 1: Figure S1.** Receiver operating characteristic curve and area under the curve for (A) CD68+ cells/HPF, (B) CD163+ cells/HPF. **Figure S2.** CD68 and CD163 expression in DLBCL. (A) Frequencies of CD68 expression level in IPI score = 0–1, 2–3, and 4–5, (B) Frequencies of CD163 expression level in IPI score = 0–1, 2–3, and 4–5.

## Data Availability

The datasets used and/or analyzed during the current study are available from the corresponding author on reasonable request.

## Abbreviations

AMC: Absolute monocyte count
CHOP: Cyclophosphamide, hydroxydaunorubicin, vincristine, prednisone
CI: Confidence interval
ECOG-PS: Eastern Cooperative Oncology Group performance status
HR: Hazard ratio
IPI: International Prognostic Index
LDH: Lactate dehydrogenase
OS: Overall survival
PFS: Progression-free survival
R-CHOP: Rituximab-cyclophosphamide, hydroxydaunorubicin, vincristine, prednisone
