# Supplementary material for: Tumor-associated macrophages predict prognosis in diffuse large B-cell lymphoma and correlation with peripheral absolute monocyte count
Source: BMC Cancer. 2019 Nov 6;19:1049. doi: 10.1186/s12885-019-6208-x (PMC6836332; doi:10.1186/s12885-019-6208-x)
Supplement: Supplementary file 1 — Additional file 1: Figure S1. Receiver operating characteristic curve and area under the curve for (A) CD68+ cells/HPF, (B) CD163+ cells/HPF. Figure S2. CD68 and CD163 expression in DLBCL. (A) Frequencies of CD68 expression level in IPI score = 0–1, 2–3, and 4–5, (B) Frequencies of CD163 expression level in IPI score = 0–1, 2–3, and 4–5. [file 12885_2019_6208_MOESM1_ESM.pdf]

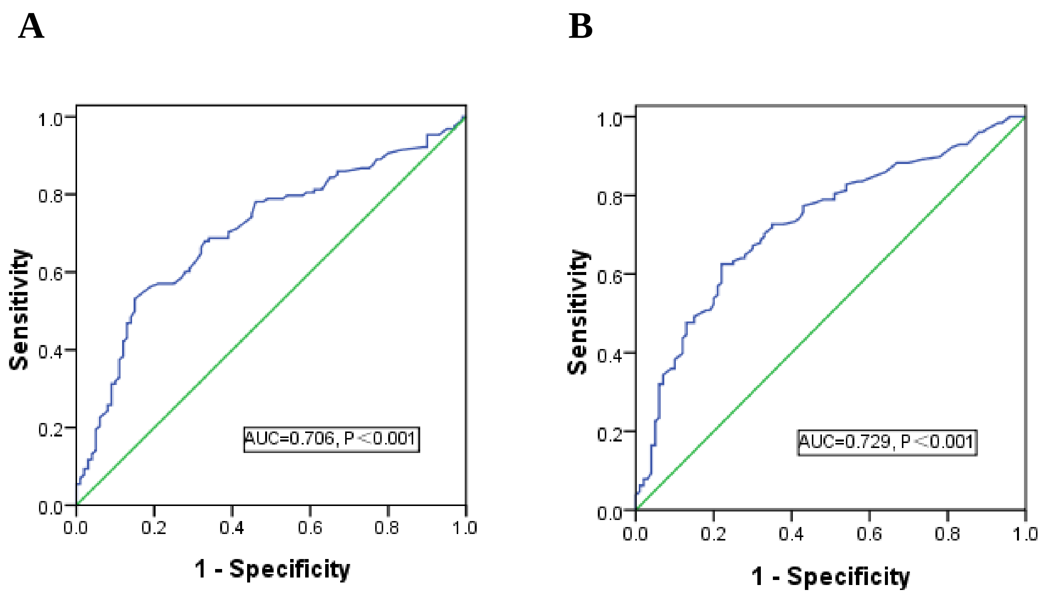

Fig. S1 Receiver operating characteristic curve and area under the curve for (A) CD68+ cells/HPF, (B) CD163+ cells/HPF.

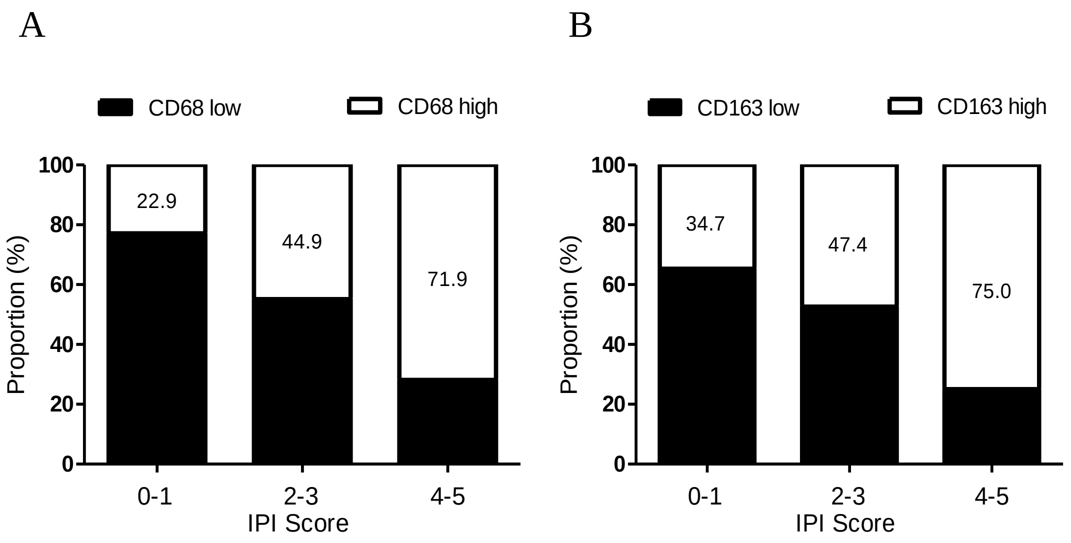

Fig. S2 CD68 and CD163 expression in DLBCL. (A) Frequencies of CD68 expression level in IPI score=0-1, 2-3, and 4-5, (B) Frequencies of CD163 expression level in IPI score=0-1, 2-3, and 4-5.
